# Simulation and Implementation of a Morphology-Tuned Gold Nano-Islands Integrated Plasmonic Sensor

**DOI:** 10.3390/s140610497

**Published:** 2014-06-13

**Authors:** Jayan Ozhikandathil, Muthukumaran Packirisamy

**Affiliations:** Optical Bio-Microsystems Laboratory, Department of Mechanical and Industrial Engineering, Concordia University, Montreal H3G 1M8, Canada; E-Mail: pmuthu@alcor.concordia.ca

**Keywords:** LSPR, gold nano-islands, biosensor, FDTD

## Abstract

This work presents simulation, analysis and implementation of morphology tuning of gold nano-island structures deposited by a novel convective assembly technique. The gold nano-islands were simulated using 3D Finite-Difference Time-Domain (FDTD) techniques to investigate the effect of morphological changes and adsorption of protein layers on the localized surface plasmon resonance (LSPR) properties. Gold nano-island structures were deposited on glass substrates by a novel and low-cost convective assembly process. The structure formed by an uncontrolled deposition method resulted in a nano-cluster morphology, which was annealed at various temperatures to tune the optical absorbance properties by transforming the nano-clusters to a nano-island morphology by modifying the structural shape and interparticle separation distances. The dependence of the size and the interparticle separation distance of the nano-islands on the LSPR properties were analyzed in the simulation. The effect of adsorption of protein layer on the nano-island structures was simulated and a relation between the thickness and the refractive index of the protein layer on the LSPR peak was presented. Further, the sensitivity of the gold nano-island integrated sensor against refractive index was computed and compared with the experimental results.

## 1. Introduction

The development of novel nano-fabrication technologies has attracted significant attention because of the plasmonic properties of nanomaterials and the feasibility of exploiting them for the label-free detection of biological and chemical substances. Surface plasmon resonance (SPR)-based sensors for the label-free detection of protein-protein interactions [1,2], DNA hybridization [3] and bacteria [4] have been widely reported. The SPR-based sensor is based on the propagation of surface plasmon waves produced by coupling of light to a thin noble metal layer by a grating coupler or near field excitation [5]. SPR-based sensors have however several drawbacks, including lower spectral resolution and the need for large equipment. Noble metal nanoparticles (NPs) such as gold and silver exhibit unique optical resonance properties in the visible and near-infrared (NIR) regions of the electromagnetic spectrum, and they have been proven to be useful for label-free detection [6–9] and feasible to integrate in a microfluidic device for the development of lab-on-a-chip devices [10]. The optical resonance behavior of NPs is commonly referred to as localized surface plasmon resonance (LSPR), which is due to the resonance response of the free electrons of the nanoparticles to the electric field of the light. One of the main advantages of LSPR-based sensor over the SPR-based sensor is that the LSPR properties of nanoparticles can be tailored to meet the requirements of different applications and the sensitivity enhanced by changing the size and shape of the nanoparticles.

LSPR-based sensors are typically fabricated using metal nanoparticles immobilized on an optically transparent substrate [11,12]. Therefore, the immobilization of nanostructures with nanoparticles of various sizes and shape is an important fabrication process for the development of nano-biosensors. Subsequently the biomolecules are adsorbed on the nanostructures and hence the biomolecular interaction can be assessed by monitoring the position and/or intensity of the LSPR band by transmission or reflection spectroscopy. The essential qualities of the nanostructures for biosensing applications are good adhesion to the substrate, ease of modification of the morphology of the nanostructures to tune the optical properties to achieve higher sensitivity, *etc.* Some of the widely employed fabrication processes for the fabrication of nanostructures are nano-sphere lithography (NSL) [13,14], vapor deposition [15], direct thermal deposition and electrochemical deposition [12], *etc.* NSL is useful for the deposition of ordered nanostructure by depositing a metallic nano-film on a self-assembled polystyrene spheres. Evaporation of gold film and annealing to yield a nanostructure film is a useful method. However the expensive instrumentation and poor adhesion are the drawbacks of this approach.

In a recent work, partially embedded gold nanoparticles were produced by annealing a gold film on a glass surface, with higher sensitivity for biosensing [16]. The convective assembly of nanoparticles using complex and expensive apparatus for the controlled deposition of nanoparticles is reported [17]. Polymer-gold (or silver) nanocomposites are also reported to be useful for the biosensing applications [18–20]. Hence the process of formation nanostructures on the substrate with suitable LSPR band is an important task in the development of nano-biosensor. The gold nano-islands obtained by the proposed low-cost convective assembly and annealing have been already demonstrated for the detection of growth hormone in a microfluidic chip in our previous work [21].

In the present work, 3D Finite-Difference Time-Domain (FDTD) simulation of gold nano-islands and nano-clusters and a comparison with the experimental results is reported. The utility of the FDTD technique for the simulation of gold nanospheres [22], nano-islands [16] and gold nanorods [23] has been widely demonstrated. The fabrication process of the nano-islands morphology including the intermediate stages of formation of nano-islands from nano-cluster morphology and the changes of optical properties are explained. The proposed method is an uncontrolled and simple convective assembly, which resulted in gold aggregates with a wide absorbance spectrum spreading in the visible and near-infrared regions of light. The morphology of gold aggregates is not useful for biosensing because of their wide resonance peak, therefore the modification of the morphology for the desired optical resonance property is achieved by a post-deposition annealing process. The tuning of morphology is analyzed by 3D FDTD simulation. In the simulation, nano-islands are approximated to hemispheres as they resemble a hemi-spherical structure, as we can observe in the SEM images. The optical resonance of the nano-cluster is investigated with four hemispheres separated by a distance “*d*”. The values of “*d*” is varied to study the nano-cluster effect, the distance “*d*” is in fact varying during the post-deposition annealing process. The modification of LSPR of the gold-hemisphere was simulated with a protein layer of certain refractive index and thickness. In addition, the effects of thickness and refractive index on the LSPR properties were also investigated. The refractive index sensitivity of the gold-nano hemisphere was estimated at 89.749 nm/RIU and compared with the experimental result of 62.879 nm/RIU.

## 2. Localized Surface Plasmon Resonance

Localized surface plasmon resonance (LSPR) is due to the surface plasmon waves produced from the collective oscillations of electrons by illuminating the nanoparticles. The oscillation of electrons in metal is described by the dielectric function as by the Drude-Lorentz [24] model as:

(1) $$\varepsilon =\varepsilon_{L}-\frac{\omega_{p}^{2}}{\omega^{2}+i\gamma \omega }+\frac{\nabla \in \omega_{p}^{2}}{-\omega^{2}+j\gamma \omega +\omega_{0}}$$

where *ε_L_* is the dielectric constant for the lower wavelengths, *ω_p_* is the plasma frequency of the free electrons, *ω* is the angular frequency of the light illuminating to the nanoparticles, *ω*_0_ is the resonance frequency and *γ* is collision frequency of the electrons present in the bulk materials. In the case of nanoparticles, as the size of the nanoparticles is much smaller than ∼10 to 100 nm, the wavelength of the light, the oscillation of the electrons can be approximated to a dipole oscillation, therefore, the collective oscillation of electrons due to electric field can be described by dipolar polarizability [25] *α*:

(2) $$\alpha =\left(1+s\right)\varepsilon_{0}V\frac{\left(\varepsilon -\varepsilon_{m}\right)}{\left(\varepsilon +s\varepsilon_{m}\right)}$$

where *V* is the volume of the nanoparticle, *ε_m_* is the dielectric constant of the medium and *s* is a factor which depends on the shape of the nanoparticles. From Equation (2), we can see that the polarizability *α* is maximum when real part of the *ε* is equal to −*sε_m_*, and the *ω_p_* satisfying this condition is the localized surface plasmon frequency of the particle. Therefore, from Equations (1) and (2), we can see that LSPR property depends on the bulk plasma frequency *ω_p_*, the geometry factor *s* and the medium where the nanoparticle is placed (*ε_m_*).

The surface plasmon results in a strong confinement of the electric field in the surface of the nanoparticles, therefore, if two nanoparticles are brought together, the near-fields of two nanoparticles will interact and the electric field experienced on each particle is a resultant field. Due to the interaction of the near-fields, the plasmon resonance of two NPs gets coupled with the influence of neighboring particles resulting in a modulated LSPR band.

In this work, a novel method of manufacturing nano-island structures on glass is discussed. The nano-cluster morphology produced by the convective assembly was transformed to nano-islands by post-deposition annealing. The effect of annealing temperature on the morphological transformation and the optical absorbance property was characterized by SEM and UV-Visible spectroscopy, respectively. The nano-island morphology was simulated with a gold nano-hemisphere model and the optical property of the nano-clusters is analyzed by 4-gold hemisphere models in FDTD. The sensitivity of the morphology to refractive index was simulated and compared with the experimental results.

## 3. FDTD Modeling

The FDTD method solves Maxwell's differential equations by discretizing using the central difference in space and time and numerical solving. Maxwell's equations relate the temporal change of electric field E on the spatial change of magnetic field (H = B/μ), and vice versa.

FDTD uses a second order finite centered approximation to the space and time derivatives in Maxwell's curl equations to get a discrete electromagnetism. In the FDTD method, an orthogonal cubic spatial grid called Yee unit cell is defined and hence the electric and magnetic field components are computed at each cell at time instants delayed by the half sampling time steps [26]. The material can be modeled by specifying its characteristics at every cell [27].

A commercially available software, RSoft FullWAVE, is used for the FDTD simulation of the gold nano-island structures. In the FDTD modelling, the gold nano-island is approximated to a hemisphere as seen in the SEM images (Section 4) where the nano-islands resemble a hemisphere. As the morphology tuning is the major focus in the present work, the effects of interparticle coupling distance, size of nano-islands and biosensing potential are investigated by estimating the LSPR properties of the nano-islands. The perfectly matched layer (PML) [28] boundary condition is used on the boundaries of the simulation domain, that is the boundary walls of the simulation domain are conductive to both the magnetic and electric fields, hence the light is completely absorbed on the boundary without any reflections.

**Figure 1.**
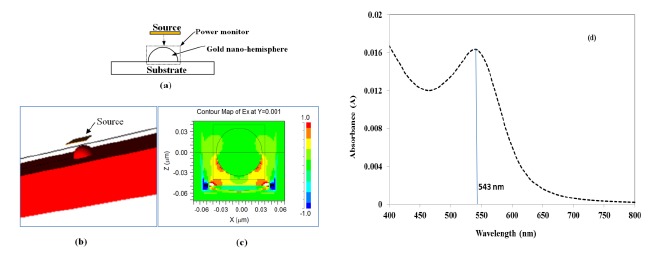
(**a**) Schematic of the model (**b**) 3D-FDTD model of the gold nano-hemisphere (**c**) electric field distribution estimated by FDTD in the gold nano-hemisphere and (**d**) optical absorbance spectrum of the gold nano-hemisphere by FDTD simulation.

Figure 1a shows a schematic of the model, wherein a gold nano-hemisphere was excited by a source with plane waves originating from a square launch pad with a transverse electric field. A pulse excitation type is used to excite the gold nano-hemisphere. The gold nano-hemisphere was kept in a power monitor to monitor the total power absorbed to the nano-hemisphere. Figure 1b shows the 3D view of the gold nano-hemisphere modeled in the Rsoft FullWAVE. The electric field distribution on the gold nano-hemisphere simulated by FDTD is shown in Figure 1c. By using the Fast Fourier Transform (FFT) algorithm, the absorbance spectrum of the gold nano-hemisphere was computed. As the simulation domain is discretized into small elements called grids or mesh, the size of the mesh decides the accuracy of the model. Large computational resources and long times are required for the simulation with smaller mesh elements, hence a mesh convergence study is carried out to find the optimum mesh size, which was found to be 1 nm.

Figure 1d shows the absorbance spectrum computed by FDTD. The diameter of the gold nano-hemisphere was set at 70 nm. The size of the nano-island was chosen at 70 nm as the proposed process of morphology tuning results in a nano-island morphology having an average size of 70 nm as explained in reference [21] and also briefly in Experimental Section (Section 4). The simulation shows an optical absorbance spectrum having a LSPR peak at 543 nm.

### 3.1. Dependence of the Size of Gold Nano-hemisphere on the Optical Absorbance Spectrum

**Figure 2.**
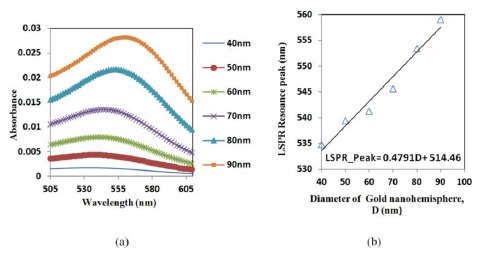
(**a**) Optical absorbance spectrum of gold nano-hemisphere (**b**) a red-shift of peak wavelength against the size of the gold nano-hemisphere.

A hemisphere was simulated to establish a relation between the size and the resonance peak of the gold nano-hemisphere. The simulation shows that the increase in the diameter (D) of the gold nano-hemisphere results in a gradual shift of optical absorbance peak towards the higher wavelength (Figure 2a).

The spectrum showed an increase in peak wavelength and in the intensity of the LSPR spectrum against the diameter (D). Moreover the spectrum is found to be widening as the size of the hemisphere increases. The diameter of the gold nano-hemisphere is increased from 40 to 90 nm. A linear trend for absorbance peak wavelength is observed as shown in the Figure 2b. A similar trend is observed in the case gold nanosphere [29] and nanorods [30] in the literature.

### 3.2. Modeling of Morphology Transformation

**Figure 3.**
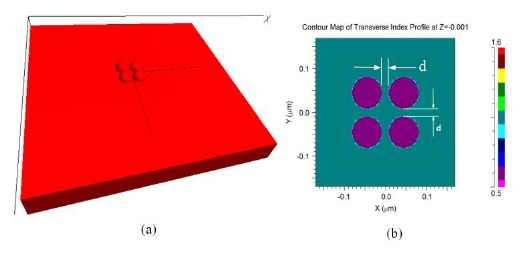
(**a**) FDTD model of the four hemisphere morphology (**b**) refractive index distribution of the FDTD model from the top of the model.

The FDTD model used for the investigation of morphological transformation is shown in Figure 3a. The model consists of four gold nano-hemispheres separated by distance *d* (Figure 3b). The diameter (D) of the hemisphere was 70 nm. The refractive index distribution of the model is shown in Figure 3b. All four hemispheres were kept in a single power monitor to investigate the resultant LSPR band. As explained before, the structure was excited with a pulse and by using the FFT, the LSPR spectrum was obtained. The effect of the separation distance (*d*) for four gold nano-hemispheres and the interparticle coupling effects on the LSPR property of the morphology are studied using this model. The spacing *d* was varied equally in both directions.

**Figure 4.**
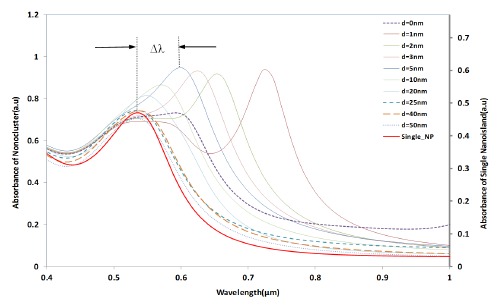
LSPR spectrum of the gold nano-hemisphere against the particle separation distance *d*.

Figure 4 shows the simulation results of the interparticle coupling effects on the LSPR property. The plasmon shift (Δλ) is the shift of the peak wavelength of the LSPR spectrum of the four nano-hemispheres from the peak wavelength of the single hemisphere from a certain separation distance *d*. The separation distance *d* of the nanostructures is increased from 0 nm to 50 nm and the LSPR spectrum is computed for each *d*. When the particles were touching each other (*d* = 0), the optical absorbance spectrum was a wide band as shown in Figure 4. When the *d* was increased to 1 nm, a dramatic change in LSPR band is observed, that is, two clearly defined bands were obtained, one wide band similar to that of touching structure and a narrow peak at 725 nm. When the particles are too close (*d* ≤ 5 nm) or touching each other (*d* = 0), gold hemispheres interact through the strong coupling of electric fields to each other, which alter the extinction spectra and hence a resultant spectra having dual peaks is observed. When the *d* was further increased, the band at higher wavelength was found to be moving towards the lower wavelength and the wide band observed in the lower wavelengths was found to be slowly vanishing. When the *d* was increased to 25 nm or above, only a single band same as that of a single gold nano-hemisphere is observed. The LSPR spectrum of the single gold hemisphere is also presented in the Figure 4.

The resultant LSPR band arises from the near-field coupling between the particles. The simulation demonstrates that the near-field coupling of the gold nano-hemisphere is negligible when the separation distance *d* is 25 nm or above, and the hemispheres are completely isolated from the near-field coupling.

**Figure 5.**
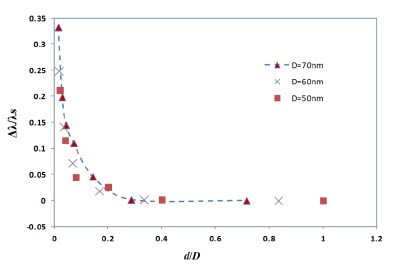
The influence of spacing on near field coupling strength between four gold nano-hemispheres.

It has been already observed that the plasmon shift decays almost exponentially against the interparticle separation distance for the case of two metal nano-spheres [31,32]. In the present study, the FDTD simulation with four gold nano-hemispheres also exhibited an exponential decay in the plasmon shift against the separation distance as shown in Figure 5. The simulations carried out for different diameters of the nano-hemispheres resulted in almost the same exponential trend. That means, the near-field coupling strength Δλ/λs falls almost exponentially over a distance of 0.3 times the diameter of the nano-islands, regardless of the size of the islands. Therefore, the interparticle coupling effects of smaller islands in the annealed morphology (Section: 4) contributes less in the resultant LSPR band as compared to larger nano-islands.

### 3.3. Sensitivity of LSPR of the Gold Nano-hemisphere to Adsorbing Protein Layer

**Figure 6.**
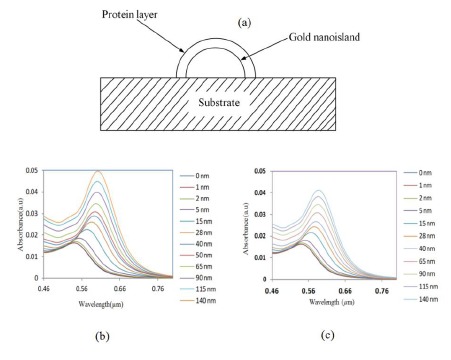
(**a**) Schematic of biosensor with gold hemispheres and a protein layer, change of LSPR spectrum against the thickness of the protein layer with a refractive index of (**b**) 1.5 and (**c**) 1.4.

It is important to investigate the sensitivity of the LSPR peak to the change in refractive index for the biosensing process. In biosensing, the analytes bind to the gold nanostructure and the refractive index of the environment of the gold changes, resulting in a shift of the LSPR band. For that, a model composed of a gold nano-hemisphere with a sensing layer equivalent to a protein layer having certain refractive indices, covering the whole surface of the nanostructure was used. Figure 6a shows the schematic representation of the model of a gold nano-hemisphere with a layer of protein.

The thickness and the refractive index of the protein layer could affect the optical absorbance property of the nanostructure, hence the effects of both the thickness and the refractive index were investigated. First, the refractive index was kept constant and the thickness of the protein layer was varied from 0 to 140 nm, subsequently the simulation was repeated for various refractive indices. Figure 6b and c show the change of LSPR spectrum of the gold-nano hemisphere against the thickness of the protein layer for the refractive index of 1.5 and 1.4, respectively.

**Figure 7.**
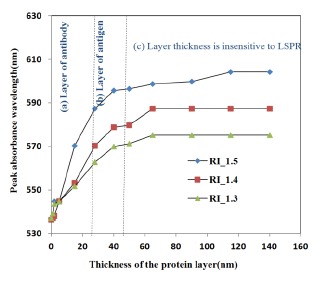
Variation of LSPR peak against the thickness of the protein.

The refractive index was varied from 1.3 to 1.5 as most proteins have a refractive index in this range [33]. The results presented in Figure 7 show that the shift of absorbance peak is saturating when the thickness of the protein layer is equal to or greater than 60–70 nm. Hence, the gold nano-hemisphere is suitable for sensing surface-assisted phenomena, which is attractive for the specific detection of antigen-antibody interactions.

In Figure 7, three protein layer thickness regions could be identified. Since the size of large bimolecules is between 10 and 20 nm [33], the protein layer of antibodies adsorbing on the gold hemisphere for a sandwiched immunoassay appears in region (a) of Figure 7. Then the antigen is added to the antibody layer, and the total thickness is between 40 and 50 nm (region (b) of Figure 7). The region above 60–70 nm of Figure 7 is insensitive to the thickness of the bio-layer. Therefore the simulation results show that gold nano-hemisphere is highly suitable for the detection of biomolecules, including large biomolecules such as proteins.

## 4. Manufacturing of Nano-islands on a Substrate

**Figure 8.**
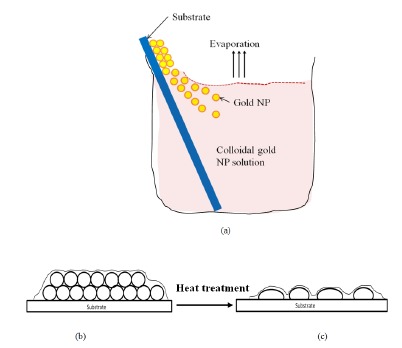
Fabrication strategy of the gold nano-island structure on a substrate. (**a**) angled convective assembly [21] (**b**) schematic of gold aggregates and (**c**) schematic of gold nano-islands structure.

The process of formation of widely separated nano-islands from the nanocluster is explained in [21]. In the present work, the dependence of the morphology and optical property of the nanostructure on annealing temperature is explained. The strategy designed for the formation of widely separated nano-islands is illustrated in Figure 8. It is relatively easy to form a multilayer of nanoparticles on the substrate in an unorganized format. For that, the particles need to be strongly adsorbed to the substrate from a colloidal suspension. Since the process of preparing a gold colloidal solution is an easy and low cost process, the gold colloidal solution of spherical gold nanoparticles were prepared by reducing chloroauric acid by sodium citrate using Turkevich's method [34]. The nanoparticles from the colloidal suspension were adsorbed on the substrate by using a novel angled convective assembly technique as shown in Figure 8a. The nanoparticles from the colloidal suspension were driven to the substrate and adsorbed upon evaporating the solvent. Even though the proposed angled convective assembly is not a precise or controlled process, it is simple and useful for the formation of aggregates or multilayers of gold nanostructures as shown in Figure 8b. Subsequently, by using heat treatment, morphological transformation of the aggregates into nano-islands is achieved.

The glass substrate was cleaned with a soap solution, deionized water, then rinsed with acetone, dried and rinsed with 2-propanol. Then the substrate was silanized and washed in toluene and heated in an oven at 100 °C for 1 h before the deposition process. For the silanization, the substrates were soaked for 1 h in 1% 3-(mercaptopropyl)trimethoxysilane (MPTMS) in ethanol. The glass substrate was immersed in the gold colloidal solution at an angle of approximately 30° in vials and kept in the oven at temperatures between 60 and 80 °C for 1–2 days, until the whole amount of gold was transferred to the substrate as shown in Figure 8b. Then the nanocluster morphology was annealed at various temperatures to study the changes of morphological and optical properties.

**Figure 9.**
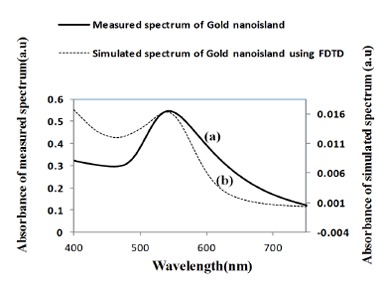
(**a**) Measured LSPR spectrum (**b**) simulated LSPR spectrum of the gold nano-hemisphere.

**Figure 10.**
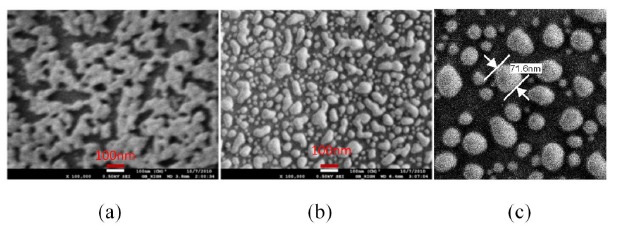
SEM micrographs of (**a**) nano-cluster obtained after deposition (**b**) annealed at 400–450 °C (**c**) annealed at 550–600 °C [21].

Figure 9 shows a comparison of the optical properties of widely separated nano-islands of average diameter of 70 nm with the simulated spectrum. Figure 9b shows the absorbance spectrum computed by FDTD for a gold nano-hemisphere of 70 nm diameter. The measured and the simulated LSPR peaks are at 545 and 543 nm, respectively and the shape of both of the spectra was found to be slightly different. The size of the nano-islands on the structures annealed at 550 °C (Figure 10c) varies from 10 to 100 nm while the modeling was carried out with a gold hemisphere of 70 nm. The size distribution of the nano-islands could contribute to the difference in the shape of the spectrum in Figure 9. The simulation results show that the approximation of a gold nano-hemisphere by 70 nm diameter, with widely separated nano-islands morphology is closer to reality.

The nano-cluster sample had a dark blue color. The morphology of the deposition is investigated by scanning electron microscopy (SEM). Figure 10a shows the SEM micrograph of the sample after the deposition. As expected, the uncontrolled convective assembly resulted in a nano-cluster morphology. The sample was annealed at various temperatures to investigate the morphological transformations. When the sample was heat treated, its color turned red.

Figure 10b shows the morphology obtained after annealing at 400 to 450 °C for 1 h. A dramatic change in morphology is observed upon annealing and the particle size ranges from 10 to 100 nm with an average separation distance of 10 nm. During the annealing, as the melting point of nanostructures is much lower than its bulk form, the nano-cluster is melting and due to the high surface tension of the molten metal, the cluster morphology transforms into a droplet-like nano-island morphology. When the annealing temperature was further increased to 550–600 °C, the separation distance between the nano-island have been found to increase as shown in Figure 10c. The morphology is composed of nanoparticles of sizes ranging from 10 to 100 nm and they are widely separated each other. Around 60 to 70% of the particles in an area of 1 μm^2^ were found having a size between 40 and 80 nm with an average separation distance of 50 nm, when the sample was annealed at above 550 °C. The adhesion of the nano-islands to the glass substrate is found to be strong and hence they are suitable for biosensing with repeatable results as demonstrated in our previous work [21].

**Figure 11.**
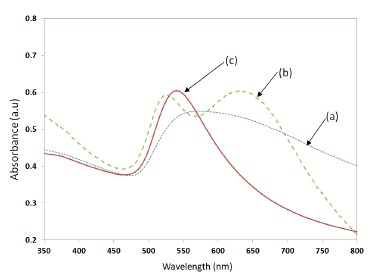
UV-Visible absorbance spectrum of (**a**) non-annealed sample (**b**) samples annealed at 400–450 °C, and (**c**) samples annealed at 550–600 °C.

The LSPR property of the samples was measured by UV-Visible spectrophotometer (Perkin Elmer, LABMDA 650). The UV-Visible absorbance spectrum measured for the three samples shown in Figure 10, is presented in Figure 11. The UV-Visible spectrum of the nano-cluster morphology obtained after the convective assembly was a wide band extending from 475 nm to 675 nm as presented in Figure 11a. The annealing of sample at 400–450 °C resulted in an LSPR band with two peaks as shown Figure 11b. When the samples were annealed at temperatures in the range of 550–600 °C, a single peak was obtained as shown in Figure 11c. A batch of 20 samples annealed at 550 °C for 1 h had a resonance peak at 545 ± 10 nm. The adhesion of the NPs with the substrate was found to be less good for annealing temperatures around 550 °C. To increase the adhesion, the annealing temperature was further increased to 600 °C and annealing was carried out for 20 h, which resulted in a nano-island morphology with very strong adhesion and a more stable resonance peak at 545 ± 5 nm.

The trend observed in Figure 4 was also observed in the experimental results given in Figure 11, wherein, when the samples were annealed at 400–450 °C, a closely packed nano-island morphology is obtained. Because of the strong near field coupling effects of closely packed nano-islands, the resultant LSPR band was found to have dual peaks (Figure 11b). Similar dual peaks were also observed in our previous work with samples deposited from a gold solution of lower concentration [21]. When the annealing temperature was increased to 550–600 °C, the peak in the higher wavelengths vanished and only one band is obtained due to the change in morphology and spacing (*d*) similar to the results in Figure 4.

## 5. Refractive Index Sensitivity of Gold Nano-Hemispheres

**Figure 12.**
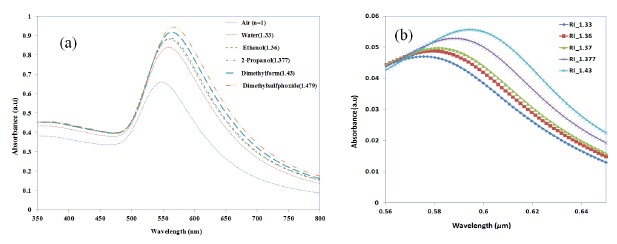
Shift of LSPR band in various solvents (**a**) measured and (**b**) simulation.

In order to assess the suitability of the nano-island-integrated platform for biosensing, the sensitivity of the nano-islands to the change in refractive index of the sensing layer has to be determined. Therefore the change of LSPR peak against the change of refractive is measured by using solvents with known refractive indices. Annealed samples were placed in a cuvette filled with a solvent and placed in the light path of the UV-Visible spectrophotometer. Herein, as the sample is placed in the bulk liquid the thickness of refractive index layer is insensitive to the LSPR band. De-ionized (DI) water with a refractive index of 1.33 was used as the reference solvent. The shift of LSPR (Δλ) for solvents was measured with reference to the LSPR peak corresponding to DI water. The LSPR spectrum measured for various solvents are shown in Figure 12a.

From Section 3.3, we have seen that the LSPR peak is not sensitive to the thickness of the protein layer when the thickness was greater than 60 nm, but only to the refractive index. Therefore, in order to investigate the refractive index sensitivity of gold nano-hemispheres, the hemispheres with a diameter of 70 nm were simulated with a sensing layer of 140 nm thickness and with different refractive indices. The refractive indices of the solvents used in the experiments are used in the FDTD model. Figure 12b shows the change of LSPR observed from the FDTD simulation for various solvents.

The refractive index sensitivity can be assessed using the relation between LSPR shift (*Δλ*) and the change in refractive index (*Δn*). Where *Δλ* and *Δn* are defined as:

(3) $$\Delta \lambda =\lambda_{m}-\lambda_{w}$$

(4) $$\Delta n=n_{m}-n_{w}$$

where *Δλ* is the change in the absorbance peak wavelength, *λ_m_* is the absorbance peak wavelength corresponding to the medium with a known refractive index, *λ_w_* is the absorbance peak wavelength corresponding to de-ionized water, *Δn* is the difference between the refractive indices of the solvent and that of water, *n_m_* is the refractive index of the solvent and *n_w_* is the refractive index of water.

**Figure 13.**
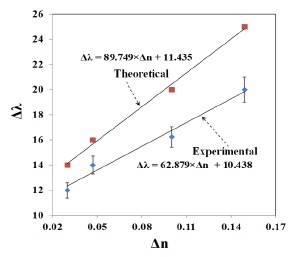
Measured and simulated variation of LSPR shift against the change in refractive indices.

The variation of LSPR shift (*Δλ*) against the change of refractive index (*Δn*) is shown in Figure 13. The error bar given in the experimental results corresponds to the standard deviation of measurements taken from 10 samples (the data used was from reference [21]). The LSPR sensitivity against refractive index, as measured by the slop of the graph shown in Figure 13 is 62.879 nm/RIU. The sensitivity predicted by FDTD simulation is 89.749 nm/RIU, as shown in Figure 13. The slight disagreement between the simulated and the measured sensitivity is due to the approximation of the nano-islands morphology with a single gold nano-hemisphere of 70 nm diameter.

There are several works in the literature on the fabrication and simulation of nanoparticle-based sensors. An array of nano-rods was fabricated using e-beam lithography and the refractive index sensitivity was 140 nm/RIU [35]. The spherical gold nanoparticles deposited using layer by layer deposition showed an enhanced sensitivity of 240 nm/RIU. Though the refractive index sensitivity of our method is lower than the other methods, our method has several advantages, including the simplicity in fabrication, low cost and suitability for integration with the microfluidic based biosensor as we demonstrated in our previous papers [21,36], which shows that the proposed nano-island-based sensor has higher sensitivity towards larger biomolecules such as growth hormone.

## 6. Conclusions

FDTD modeling of a morphological transformation of nano-clusters into nano-islands and its LSPR sensitivity is presented in this paper. Formation of a nano-island morphology by a simple and low-cost convective assembly followed by a post-deposition annealing is discussed. The nano-cluster is modeled with four gold nano-hemispheres, and the separation distance between them is varied to study coupling effects and the resultant LSPR spectrum. It is found that the near-field coupling strength falls exponentially for a spacing of 0.3 times the diameter of the nano-islands. Hence the field coupling effects due to smaller particles in the nano-island morphology is considered negligible. The biosensing potential of the gold nano-islands is investigated with an equivalent biosening layer having different thickness and refractive indices. The effects of thickness of the protein layer on the LSPR properties are found negligible when the thickness was greater than 60 nm, making the nano-island morphology highly suitable for the detection of antigen-antibody interactions, even for the larger molecules such as proteins and peptides. The LSPR sensitivity against refractive index sensitivity was investigated experimentally and theoretically. The theoretical sensitivity is 89.749 nm/RIU, which is found to be greater than the experimentally obtained sensitivity of 62.879 nm/RIU. The simulation is carried out with a simplified model of the nano-island morphology, in which a single gold hemisphere having the diameter of 70 nm is approximated with nano-island morphology obtained with the annealing temperature of 550 °C. However, the sensitivity analysis shows that the simulation and experimental results have same trend.
